# Unraveling ferroptosis in osteogenic lineages: implications for dysregulated bone remodeling during periodontitis progression

**DOI:** 10.1038/s41420-024-01969-6

**Published:** 2024-04-26

**Authors:** Yiqi Tang, Sihui Su, Rongcheng Yu, Chenxi Liao, Zhili Dong, Chengyao Jia, Vicky Yau, Liping Wu, Weimin Guo, Jinxuan Zheng

**Affiliations:** 1 Hospital of Stomatology, Guangdong Provincial Key Laboratory of Stomatology, Guanghua School of Stomatology, Sun Yat-sen University, Guangzhou, 510055 PR China; 2 Department of Oral and Maxillofacial Surgery, University at Buffalo, Buffalo, NY 14214 USA; 3 Department of Orthopedic Surgery, Guangdong Provincial Key Laboratory of Orthopedics and Traumatology, First Affiliated Hospital, Sun Yat-sen University, Guangzhou, 510080 PR China

**Keywords:** Cell death, Periodontitis, Chronic inflammation, Experimental models of disease

## Abstract

Periodontitis is a highly prevalent disease characterized by inflammation and destruction of tooth-supporting tissues that leads to tooth loss in extreme situations. Elucidating the underlying mechanisms of periodontitis pathogenesis and progression will establish the groundwork for developing effective treatment strategies. Recently, evidence concerning the role of ferroptosis in periodontitis progression has emerged. Osteogenic lineage cells are key regulators of bone remodeling. Osteogenic cell death, as observed in experimental periodontitis models, disrupts the balance between bone resorption and bone formation. However, whether the osteogenic lineage undergoes ferroptosis during periodontitis and the corresponding effect on periodontitis progression remain elusive. Here, we investigated cell-specific ferroptosis within the alveolar bone in a murine periodontitis model. Through immunofluorescence double staining and immunohistochemistry, we identified ferroptotic osteocytes and osteoblasts in inflammatory alveolar bone. Next, in vivo administration of erastin or liproxstatin-1 was conducted to either induce or inhibit ferroptosis, respectively. Severe bone resorption and inflammation, accompanied by increased osteoclast formation and impaired osteogenic potential were detected following ferroptosis activation. Subsequently, we carried out in vitro experiments on osteocytes and further verified that ferroptosis enhanced the osteocytic expression of RANKL and IL-6. These findings suggest that ferroptosis occurring within the osteogenic lineage acts as a catalyst in the progression of periodontitis by stimulating osteoclastogenesis through the secretion of inflammatory cytokines and inhibiting osteoblastic function, providing insights into ferroptosis-induced alterations in microenvironment-based intercellular communication. Ferroptosis is a promising target for controlling inflammation and preventing bone resorption in periodontitis.

## Introduction

Periodontitis is the most prevalent oral disease worldwide. From 2011 to 2020, approximately 62% of dentate adults suffered from periodontitis, with severe periodontitis accounting for 23.6% [1]. Symptoms of periodontitis include gingival inflammation, attachment loss, and alveolar bone resorption. Without timely treatment, these pathological changes may eventually lead to tooth loss. During periodontitis, the prevailing inflammatory microenvironment within the periodontium is characterized by bacterial invasion, elevated levels of proinflammatory cytokines, degradation of the extracellular matrix, etc. Proper inflammatory control is beneficial for reducing alveolar bone loss. Nevertheless, existing approaches for periodontal regeneration still have limited success, rendering it crucial to prevent the progression of periodontitis at early stages. Fully unveiling the molecular mechanisms involved in periodontitis pathogenesis and progression may help in the development of novel preventive or therapeutic strategies to combat periodontitis.

Osteogenic lineage cells, including osteoblastic precursor cells, osteoblasts, and osteocytes, are key regulators of bone remodeling. Osteoblast precursors migrate from the bone marrow to bone surfaces where active bone remodeling occurs and gradually differentiate into osteoblasts, which are responsible for the secretion and mineralization of the bone matrix [2]. Additionally, osteoblasts communicate with osteoclasts by secreting soluble proteins such as RANKL, OPG, M-CSF, IL-6, and IL-8 [3–5]. Ultimately, these cells may undergo apoptosis, mature into osteocytes, or dedifferentiate into bone-lining cells [2]. Osteocytes, as primary sources of RANKL and OPG, play pivotal roles in the regulation of osteoclastogenesis [6]. Osteocytes also inhibit osteoblastic bone formation by secreting sclerostin, a negative regulator of the canonical Wnt signaling pathway [7]. During periodontitis, the imbalance between bone resorption and bone formation may result from altered conditions of osteogenic lineage cells. To date, increased cell death within the periodontium, including osteoblasts and osteocytes, has been detected in experimental periodontitis models [8–10]. The death of osteoblasts disrupts the regular osteogenic process, while osteocyte death triggers osteoclastogenesis and promotes bone loss [11]. The synchronized effect of osteoblast and osteocyte death may underlie alveolar bone resorption in periodontitis.

Several types of cell death caused by inflammation, including ferroptosis, have been reported. Ferroptosis is a nonapoptotic form of cell death driven by iron-dependent lipid peroxidation [12]. Intracellular iron is imported through membranous transferrin receptor protein 1 (TfR1) by binding to transferrin, a portion of which is stored in ferritin. An important mechanism of iron overload within cells involves excess autophagic degradation of ferritin, known as ferritinophagy, which is mediated by NCOA4 [13]. Labile iron and Fe^2+^-dependent enzymes, including lipoxygenases and cytochrome p450 oxidoreductase, are essentially required for lipid peroxidation [14]. The end product of this process, polyunsaturated fatty acid phospholipid hydroperoxide, as well as its secondary products (e.g., 4-HNE, MDA), eventually triggers cell death. Several ferroptosis surveillance systems have been identified. The system x_c_^−^/GSH/GPX4 axis is the major antioxidant system that inhibits the accumulation of lipid reactive oxygen species (ROS) and suppresses ferroptosis [12]. Erastin, a typical ferroptosis activator, blocks system x_c_^−^-mediated cystine uptake, resulting in decreased synthesis of GSH and impaired function of GPX4. Other GPX4-independent antioxidant systems also exist, such as FSP1/CoQ10, DHODH, GCH1/BH4 and the recently discovered enzymes MBOAT1 and MBOAT2 [12, 15].

Current experimental findings implicate ferroptosis in the progression of inflammatory diseases. For instance, *Mycobacterium tuberculosis* infection induces ferroptosis in macrophages [16]. Depletion of *Gpx4* in hematopoietic cells increases host susceptibility to *Mycobacterium tuberculosis* infection and promotes pulmonary necrosis [17]. Ferroptosis inhibition also ameliorates noninfectious inflammation, such as in experimental colitis [18]. In chronic periodontitis, MDA levels in the saliva and gingival crevicular fluid rise significantly [19]. Consistently, periodontitis gingiva exhibits overproduction of ROS and iron overload [20], indicating oxidative stress in the periodontal inflammatory microenvironment. Moreover, bacteria have been identified inside osteocyte lacunae in an experimental periodontitis model [21]. Previous studies have suggested that *Porphyromonas gingivalis* (*P.g.*) triggers ferritinophagy in periodontal ligament fibroblasts, leading to excessive production of ROS [22]. To date, studies have indicated the occurrence of ferroptosis in periodontitis gingiva and its correlation with the severity of the condition [20]. However, whether ferroptosis occurs within the osteogenic lineage in periodontitis and consequently affects the bone remodeling process remains to be elucidated. Here, we demonstrate for the first time that inflammatory stimulation induces ferroptosis in the osteogenic lineage, leading to subsequent secretion of osteocyte-derived proinflammatory cytokines and impairment of osteoblast activity in periodontitis. This cascade further amplifies inflammation and facilitates bone resorption. Our study provides insights into cell communication upon ferroptosis within bone tissue.

## Results

### Construction of a murine periodontitis model

**Fig. 1:**
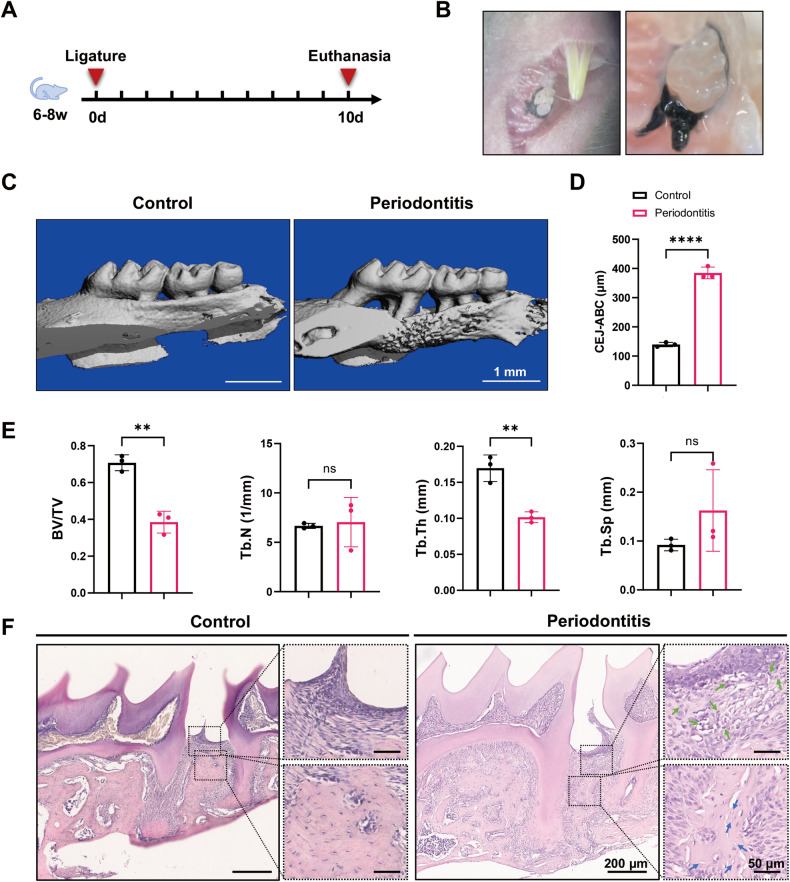
Construction of a murine periodontitis model. **A**, **B** Schematic illustration and intraoral images (captured on Day 0) of the ligature-induced murine periodontitis model. **C** Representative 3D micro-CT images of murine upper molars and alveolar bone on the control side and ligature side. Scale bar, 1 mm. **D** Quantification of the distance between the cementum enamel junction and alveolar bone crest (CEJ-ABC distance). **E** The bone volume over total volume (BV/TV), trabecular number (Tb.N), trabecular thickness (Tb.Th), and trabecular spacing (Tb.Sp) of the ROIs in the maxillary alveolar bone on the control side and ligature side. **F** Representative images of H&E-stained sagittal sections of the upper first molars on the control side and on the ligature side and magnified views of the gingiva and alveolar bone distal to the upper first molar. The green arrows indicate inflammatory cells. The blue arrows indicate empty osteocyte lacunae. Scale bar, 200 μm (panoramic view)/50 μm (magnified view). The data are shown as the means ± SDs; *n* = 3 mice; ns not significant; **P < 0.01; ****P < 0.0001.

To investigate whether periodontitis induces ferroptosis in both the gingiva and alveolar bone, we constructed a murine periodontitis model through ligature of the left upper first molar, and the right side was saved as the control. The samples were harvested on Day 10 (Fig. 1A, B). To examine the effectiveness of the ligature-induced periodontitis model, micro-CT analysis was performed on both sides of the maxilla. The vertical distance between the cementum-enamel junction and the alveolar bone crest (CEJ-ABC) significantly increased in the periodontitis group, indicating severe bone resorption (Fig. 1C, D). The bone parameters of the region of interest (ROI) were also examined, but only the BV/TV and Tb.Th of the periodontitis group significantly decreased. Tb.N and Tb.Sp did not significantly differ between the groups (Fig. 1E). For histological evaluation, we performed haematoxylin-eosin (H&E) staining of sagittal sections of the upper molars. Elevated inflammatory infiltration and increased numbers of empty osteocyte lacunae were observed in the periodontitis group (Fig. 1F).

### Ferroptosis is induced in the osteogenic lineage and gingival cells during periodontitis

**Fig. 2:**
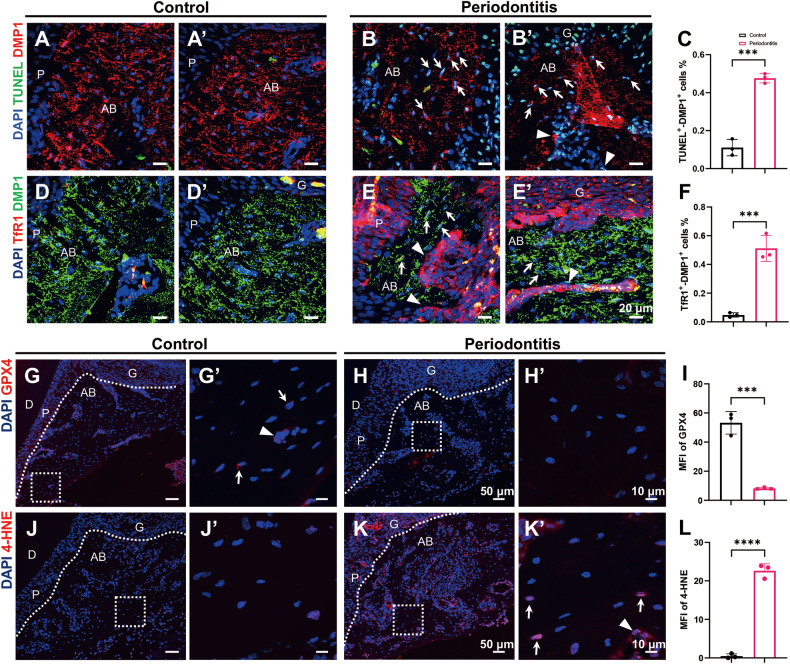
Periodontitis induces ferroptosis in osteogenic lineage cells. **A**–**B’** Representative TUNEL (green) and DMP1 (red) double-staining images of dead osteogenic lineage cells in the distal alveolar bone of the upper first molars. The white arrows indicate dead osteocytes. The white arrowheads indicate dead osteoblasts. Scale bar, 20 μm. **C** The percentage of TUNEL (+) DMP1 (+) cells among all DMP1 (+) cells was quantified. **D**–**E’** Representative images of double immunofluorescence staining of TfR1 (red) and DMP1 (green) in distal regions of the upper first molars. The white arrows indicate TfR1-positive osteocytes. The white arrowheads indicate TfR1-positive osteoblasts. Scale bar, 20 μm. **F** The percentage of TfR1 (+) DMP1 (+) cells among all DMP1 (+) cells was quantified. **G**, **H** Representative GPX4 (red) immunofluorescence images of the distal regions of the upper first molars. Scale bar, 50 μm. **G’**, **H’** Magnified views of the boxed areas in (**G**, **H**). The white arrows indicate GPX4-positive osteocytes. The white arrowheads indicate GPX4-positive osteoblasts. Scale bar, 10 μm. **I** Quantification of the mean fluorescence intensity of GPX4 in alveolar bone. **J**, **K** Representative 4-HNE (red) immunofluorescence images of the distal regions of the upper first molars. Scale bar, 50 μm. **J’**, **K’** Magnified views of the boxed areas in (**J**, **K**). The white arrows indicate 4-HNE-positive osteocytes. The white arrowheads indicate 4-HNE-positive osteoblasts. Scale bar, 10 μm. **L** Quantification of the mean fluorescence intensity of 4-HNE in alveolar bone. G gingiva, AB alveolar bone, P periodontal ligament, D dentin. The data are shown as the means ± SDs; *n* = 3 mice; ***P < 0.001; ****P < 0.0001.

Subsequently, we conducted TUNEL staining to evaluate cell death in periodontal tissues. A significantly greater number of dead cells were observed in the gingiva of the periodontitis group (Fig. S1A–C). Cell death in the osteogenic lineage was determined by double fluorescence staining for TUNEL and DMP1. There were more TUNEL- and DMP1-positive cells in the periodontitis group than in the control group (Fig. 2A–C). Cells scattered within the bone matrix were identified as osteocytes (Fig. 2B, B’ arrows), while those located along the bone surface were characterized as osteoblasts (Fig. 2B’ arrowheads).

To verify whether the cell death was ferroptosis, we detected the level of 4-HNE, a marker of lipid peroxidation [23], and the expression of ferroptosis marker genes in periodontal tissues [23]. In the periodontitis group, 4-HNE expression was significantly greater in both the gingiva and alveolar bone than in the control group (Figs. 2J–L, S1D–F, S2), and more 4-HNE-positive osteocytes and osteoblasts were also detected (Figs. 2K’and S2). TfR1 has been demonstrated to be a specific marker for ferroptosis [24]. The periodontitis group exhibited increased TfR1 expression in both the gingiva (Fig. S1J–L) and alveolar bone and enhanced TfR1 expression was detected in DMP1-positive cells by double immunofluorescence staining (Fig. 2D–F), further suggesting that osteogenic cells undergo ferroptosis in periodontitis. GPX4 is an enzyme that protects cells against oxidative damage and acts as a crucial marker for ferroptosis [25]. The results showed reduced expression of GPX4 in both the gingiva and alveolar bone by immunofluorescence staining (Figs. 2G–I, S1G–I), consistent with a lower gingival *Gpx4* level and higher *Acsl4* and *Ptgs2* levels by RT‒qPCR in the periodontitis group (Fig. S1M). Taken together, ferroptosis is induced in both the gingiva and the osteogenic lineage during periodontitis.

### Ferroptosis is associated with the progression of periodontitis in the murine model

**Fig. 3:**
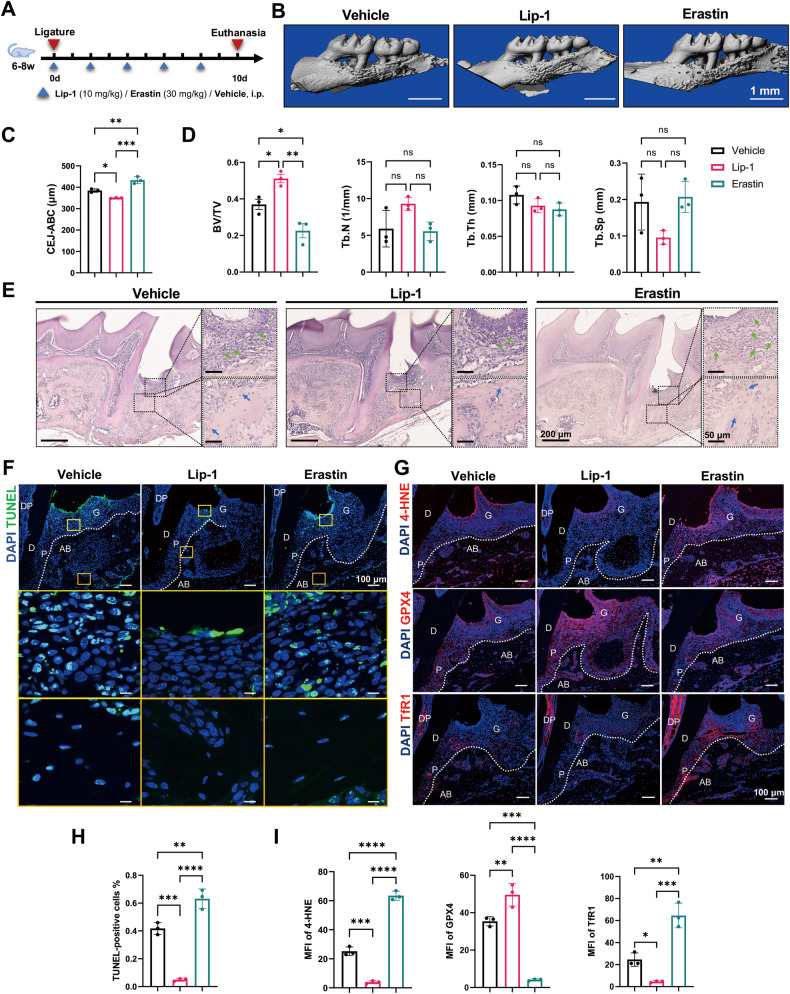
Ferroptosis is associated with periodontitis progression. **A** Schematic illustration of drug administration during ligature. **B** Representative 3D micro-CT images of murine upper molars and alveolar bone in the vehicle, Lip-1, and erastin groups. Scale bar, 1 mm. **C** Quantification of the distance between the cementum enamel junction and alveolar bone crest (CEJ-ABC distance). **D** The bone volume over total volume (BV/TV), trabecular number (Tb.N), trabecular thickness (Tb.Th), and trabecular spacing (Tb.Sp) of the ROIs in the maxillary alveolar bone on the ligature side. **E** Representative images of H&E-stained sagittal sections of the upper first molars on the ligature side and magnified views of the gingiva and alveolar bone distal to the upper first molar. The green arrows indicate inflammatory cells. The blue arrows indicate empty osteocyte lacunae. Scale bar, 200 μm (panoramic view)/50 μm (magnified view). **F** Representative TUNEL staining of dead cells in distal regions of upper first molars. Magnified views of the gingiva or alveolar bone are shown in the yellow or orange frames, respectively. Scale bar, 100 μm (panoramic view)/10 μm (magnified view). **G** Representative immunofluorescence images of 4-HNE, GPX4, and TfR1 expression in distal regions of the upper first molars. Scale bar, 100 μm. **H** The percentage of TUNEL-positive cells was quantified. **I** Quantification of the mean fluorescence intensity of 4-HNE, GPX4, and TfR1 in the alveolar bone of the vehicle, Lip-1, and erastin groups. G gingiva, AB alveolar bone, P periodontal ligament, D dentin, DP dental pulp. The data are shown as the means ± SDs; *n* = 3 mice; ns not significant; *P < 0.05; **P < 0.01; ***P < 0.001; ****P < 0.0001.

To elucidate the role of ferroptosis in periodontitis, we further administered a ferroptosis inducer (erastin), ferroptosis inhibitor (liproxstatin-1) or vehicle to the murine periodontitis model (Fig. 3A). Micro-CT analysis of the CEJ-ABC distance and BV/TV indicated that liproxstatin-1 (Lip-1) alleviated periodontitis-induced bone resorption, while erastin exacerbated this effect (Fig. 3B–D). Tb.N, Tb.Th and Tb.Sp did not significantly differ among the groups (Fig. 3D). H&E staining revealed aggravated gingival inflammation induced by erastin, while Lip-1 mitigated the inflammatory response. Moreover, inhibition of ferroptosis exhibited a protective effect against osteocyte death (Fig. 3E). To verify the effectiveness of erastin and Lip-1, TUNEL staining and immunofluorescence staining of 4-HNE, GPX4, and TfR1 were performed (Fig. 3F–I). Erastin significantly induced ferroptosis in periodontal tissue, whereas Lip-1 administration suppressed periodontitis-induced ferroptosis. The above results revealed that the induction or inhibition of ferroptosis in periodontal tissues was associated with the severity of alveolar bone resorption and gingival inflammation in periodontitis patients, suggesting that ferroptosis might play a pivotal role in bone loss during periodontitis.

### Disordered bone homeostasis followed by ferroptosis activation in periodontal tissues

**Fig. 4:**
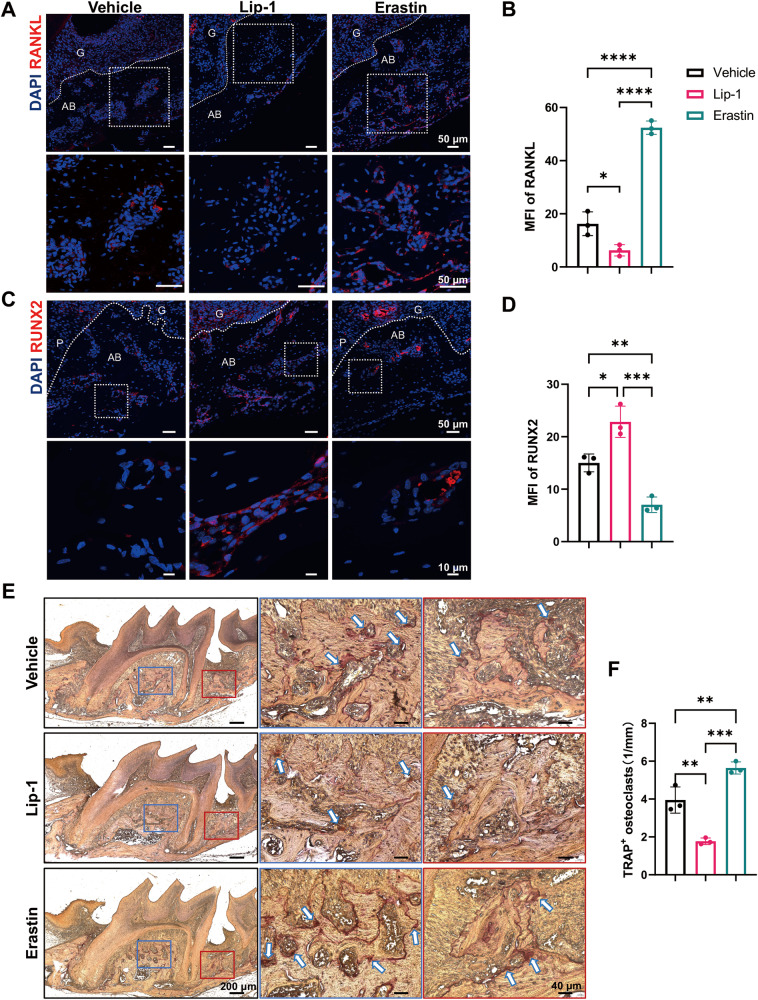
Ferroptosis activation/inactivation affects normal bone remodeling. **A** Representative immunofluorescence images showing RANKL expression in distal regions of upper first molars in the vehicle, Lip-1, and erastin groups. The lower panel shows magnified views of the boxed areas in the upper panel. Scale bar, 50 μm. **B** Quantification of the mean fluorescence intensity of RANKL in the alveolar bone. **C** Representative immunofluorescence images of RUNX2 expression in distal regions of upper first molars. The lower panel shows magnified views of the boxed areas in the upper panel. Scale bar, 50 μm (panoramic view)/10 μm (magnified view). **D** Quantification of the mean fluorescence intensity of RUNX2 in alveolar bone. **E** Representative images of TRAP staining in the vehicle, Lip-1, and erastin groups. Magnified views of alveolar bone in the interradicular region or distal alveolar bone are shown in the blue or red frames, respectively. The white arrows indicate TRAP-positive cells. Scale bar, 200 μm (panoramic view)/40 μm (magnified view). **F** Quantification of TRAP-positive cells per mm of bone surface. G gingiva, AB alveolar bone. The data are shown as the means ± SDs; *n* = 3 mice; *P < 0.05; **P < 0.01; ***P < 0.001; ****P < 0.0001.

Since bone loss is typically attributed to disrupted bone homeostasis, we further performed immunofluorescence staining for RANKL and RUNX2 to examine the osteoclastogenic and osteogenic potential. Compared with vehicle treatment, treatment with erastin significantly increased RANKL expression in the alveolar bone, while treatment with Lip-1 reduced RANKL expression (Fig. 4A, B). RUNX2 expression in the alveolar bone was upregulated by the inhibition of ferroptosis, while the activation of ferroptosis by erastin administration decreased RUNX2 signaling (Fig. 4C, D). Lip-1 reduced while erastin increased the number of TRAP-positive osteoclasts and the expression of RANKL (Fig. 4E, F). Taken together, these data suggest that ferroptosis-induced bone resorption during periodontitis is likely due to increased osteoclast activity and impaired osteogenic ability. The differential expression of RANKL among the groups indicated alterations in intercellular communication caused by ferroptosis.

### LPS-induced osteocyte ferroptosis triggers the expression of inflammatory cytokines

**Fig. 5:**
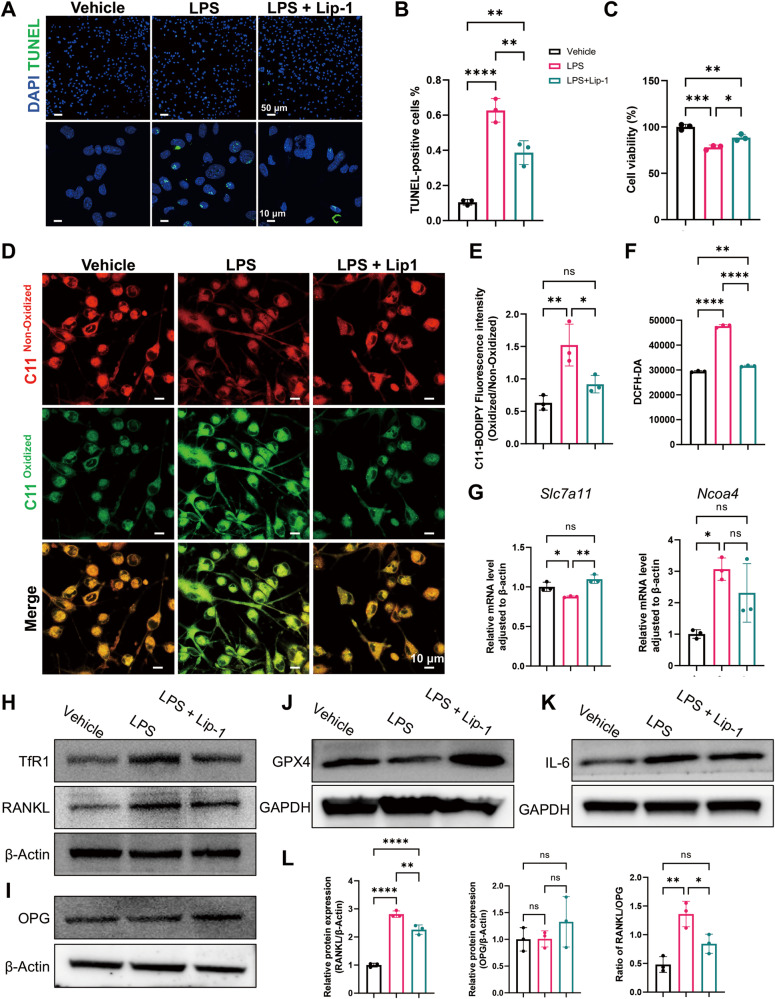
LPS-induced osteocyte ferroptosis triggers the expression of RANKL and IL-6. **A** TUNEL assay showing dead MLO-Y4 cells after treatment with vehicle, LPS, or LPS + Lip-1. Scale bar, 50 μm (panoramic view)/10 μm (magnified view). **B** Statistics of the percentage of TUNEL-positive osteocytes. **C** CCK-8 assay of treated MLO-Y4 cells. **D** C11-BODIPY staining showing nonoxidized lipids (red) and oxidized lipids (green) in treated MLO-Y4 cells. Scale bar, 10 μm. **E** Quantification of the mean fluorescence intensity of C11-BODIPY (green/red ratio). **F** Measurement of intracellular ROS levels in treated MLO-Y4 cells. **G** Relative mRNA expression of *Slc7a11* and *Ncoa4* in treated MLO-Y4 cells. **H**–**K** Western blot analysis of TfR1, RANKL, OPG, GPX4, and IL-6 expression in treated MLO-Y4 cells. **L** Quantification of the relative RANKL and OPG protein expression and the RANKL/OPG ratio. The data are shown as the means ± SDs; *n* = 3 biological replicates; ns not significant; *P < 0.05; **P < 0.01; ***P < 0.001; ****P < 0.0001.

Osteocytes play a central role in the regulation of bone homeostasis; they modulate osteoclast formation and activation mainly via RANKL/OPG and simultaneously release other inflammatory cytokines, such as IL-6 and IL-1β. Osteocytes are the main source of RANKL and our previous sections confirmed the occurrence of osteocyte ferroptosis in periodontitis (Fig. 2); thus, we selected osteocytes for subsequent experiments. To further determine whether osteocyte ferroptosis contributes to bone resorption in periodontitis, we conducted in vitro experiments by treating MLO-Y4 cells with LPS from *P.g.* (*P.g.*-LPS), *P.g.*-LPS + Lip-1, or vehicle. First, we examined whether LPS induced ferroptosis in osteocytes. TUNEL staining revealed a significant increase in cell death induced by LPS, while Lip-1 alleviated this effect (Fig. 5A, B). Additionally, CCK-8 analysis demonstrated that Lip-1 improved the decrease in cell viability induced by LPS (Fig. 5C). Intracellular lipid peroxidation was assessed using the C11-BODIPY fluorescent probe. Following LPS stimulation, MLO-Y4 cells exhibited increased lipid peroxidation, which was reversed by Lip-1 treatment (Fig. 5D, E). Concurrently, the detection of total intracellular ROS using a DCFH-DA probe yielded results consistent with those of the C11-BODIPY assay (Fig. 5F). SLC7A11 is the light chain subunit of system x_c_^−^, which mediates cellular cystine uptake and the biosynthesis of glutathione, protecting cells from oxidative stress [26]. NCOA4 facilitates ferroptosis by mediating ferritinophagy. The relative mRNA level of *Slc7a11* decreased upon LPS stimulation and was rescued by Lip-1 treatment, with *Ncoa4* showing the opposite trend (Fig. 5G). Consistently, LPS treatment led to a notable decrease in GPX4 protein expression and an increase in TfR1 expression. Administration of Lip-1 partially reversed this effect (Fig. 5H, J). Taken together, these findings demonstrate that LPS triggers ferroptosis in osteocytes in vitro.

To explore the potential mechanisms through which osteocyte ferroptosis facilitates bone resorption, we detected the protein levels of RANKL, OPG, and IL-6 in treated MLO-Y4 cells. The elevated RANKL, IL-6 levels, and the RANKL/OPG ratio upon LPS induction were reduced by the additional administration of Lip-1 (Fig. 5H, I, K, L). Current data indicate that ferroptosis upregulates the osteocytic expression of RANKL and IL-6, possibly altering the periodontal microenvironment and contributing to subsequent osteoclastogenesis and bone resorption. Additionally, the periodontitis-affected gingiva also produced more *IL-1β* and less *Opg* than the controls, as evidenced by RT‒qPCR. However, the *Rankl* level in the gingiva did not significantly differ between the periodontitis group and the control group (Fig. S1N). The large amount of RANKL expression after ferroptosis activation in the alveolar bone of the periodontitis group might be mostly attributed to the osteogenic lineage, especially osteocytes.

## Discussion

Mounting evidence has highlighted strong correlations between ferroptosis and inflammatory diseases, among which periodontitis is also indicated. The inflammatory microenvironment contains various proinflammatory cytokines and even microbial components. These cytokines, including IL-1β, IL-6, TNF-α, and IFN-γ, modulate ferritin synthesis, thereby influencing intracellular iron storage levels [27]. Activation of the IL-6-JAK2-STAT3 pathway stimulates the expression of hepcidin, which binds to ferroportin on the cell surface, impeding iron export and leading to intracellular iron accumulation [28]. Moreover, inflammation is commonly associated with oxidative stress [29]. Activation of the NF-κB pathway by agents such as LPS, TNF, and IL-1 leads to reduced expression of antioxidants such as GPX4, NQO1, and HMOX1 [27]. Hence, the inflammatory microenvironment initiates ferroptosis possibly through inducing iron overload and oxidative stress. However, our study demonstrated that more active osteoclasts lurked in the periodontitis model after ferroptosis induction, suggesting that the above mechanisms may not be suitable for osteoclasts under inflammatory conditions. Inflammation triggers osteoclastogenesis and bone resorption. Consequently, osteoclasts seem to be exempt from inflammation-induced ferroptosis, maintaining their active function throughout the process. Although oxidative stress impairs osteoblast function [30, 31], it also facilitates osteoclast differentiation [32, 33]. Inhibition of the NF-κB pathway, though it may enhance the production of antioxidants, as described earlier, suppresses osteoclast differentiation [34]. Specifically, LPS, IL-1, and TNF-α support osteoclast survival and differentiation rather than causing oxidative damage and cell death [35–37].

Within the skeletal system, DMP1 is also expressed by osteoblasts on the bone surface, osteoblast precursors at the metaphysis chondro-osseous junction, and certain stromal cells in the bone marrow in addition to osteocytes [38]. Based on our current results, we believe that the osteogenic lineage undergoes ferroptosis during periodontitis, which is consistent with the findings of a recent study [39]. Among the osteogenic lineage, osteocytes are the main sources of RANKL and OPG. Osteocytes exhibit significantly greater RANKL expression than their precursor cells when stimulated with TLR2 and TLR4 agonists [40]. RANKL-RANK signaling promotes osteoclast formation and activation, while OPG acts as a decoy receptor for RANKL that hinders this process by preventing its binding to RANK [41]. Additionally, osteocytes express other inflammatory cytokines, such as IL-1β, IL-6, IL-8, and TNF-α, under inflammatory stimulation that facilitates the bone resorption process [42].

**Fig. 6:**
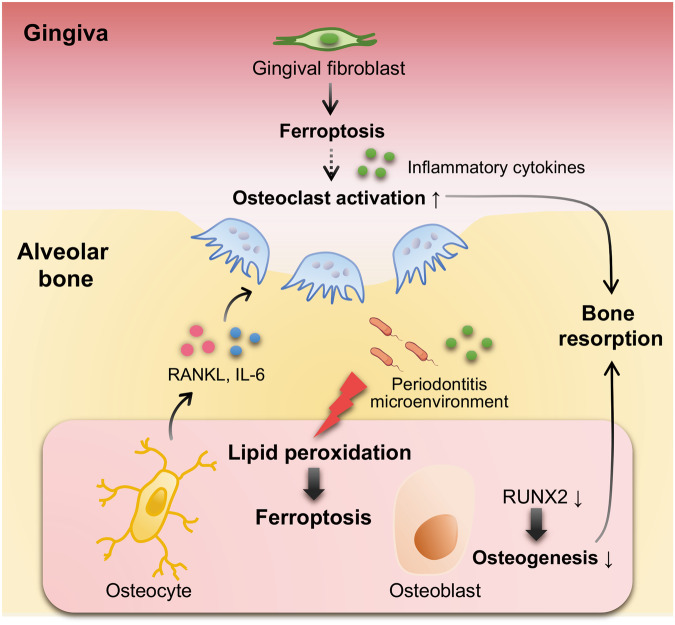
Schematic illustration of the mechanisms involving ferroptosis in the progression of periodontitis. Under inflammatory conditions, ferroptosis in the osteogenic lineage promotes periodontal bone resorption by stimulating osteoclastogenesis through the secretion of inflammatory cytokines and inhibiting osteoblastic function. Ferroptosis in gingival fibroblasts may also contribute to osteoclast formation and activation via inflammatory cytokines.

Osteocyte death likely facilitates RANKL production, as evidenced by single-cell RNA sequencing showing that dying DMP1-positive cells exhibit increased RANKL expression [43]. Bioinformatics analyses also revealed that transcription factors involved in cell death and senescence act on an osteocyte-specific intronic enhancer in the *TNFSF11* gene locus [43]. Here, we demonstrated that ferroptosis upregulates RANKL expression in osteocytes. The underlying mechanisms may be related to elevated ROS levels. A previous study revealed that increased oxidative stress and RANKL secretion concurrently occur in bone explants from osteoblast-specific autophagy-deficient mice [44]. Antioxidants such as glutathione, N-acetylcysteine, and lipoic acid rescue osteocytes from starvation-induced apoptosis and reduce their RANKL expression [45]. Similarly, oxidized lipids enhance the production of RANKL in T lymphocytes [46], suggesting a potential role for lipid peroxidation in facilitating RANKL secretion in osteocytes. Additionally, erastin-induced osteoblast ferroptosis significantly inhibits the osteogenic ability of osteoblasts [39], further contributing to bone loss. Moreover, it has been reported that the administration of the ferroptosis inhibitor Fer-1 to mice with periodontitis reduces the expression of gingival IL-6, which is a proinflammatory cytokine that increases most prominently in gingival fibroblasts during periodontitis [20]. IL-6 may indirectly contribute to bone resorption in periodontitis through interaction with IL-6 receptors on osteoblastic cells [47, 48]. Hence, it is plausible that ferroptosis of both the osteogenic lineage and gingival fibroblasts synergistically contributes to the progression of periodontitis, as illustrated in the schematic figure (Fig. 6).

In periodontitis models, bone resorption becomes obvious as early as 3 days after ligature [49]. Consistently, the number of osteoclasts in the alveolar bone, RANKL expression in gingival tissues, and RANKL-positive osteocytes all peak on Day 3 [8, 50, 51]. Whether ferroptosis contributes to early bone resorption still remains unclear since most related studies have chosen to establish 7-day or 10-day periodontitis models. However, gingival expression of GPX4 and SLC7A11 starts to decrease significantly on the first day after ligature [52]. Increased osteocyte death is also observed on Day 1 following ligature [8]. Given this information, ferroptosis may be an early event in the progression of periodontitis, and inhibiting ferroptosis promptly could be essential in clinical practice to prevent bone loss.

In conclusion, ferroptosis occurring within the osteogenic lineage catalyzes the progression of periodontitis by stimulating osteoclastogenesis through the secretion of inflammatory cytokines and inhibiting osteoblastic function, creating an osteoclastogenic-inclined periodontal microenvironment. This study offers novel insights into the mechanisms of periodontitis progression from the perspective of ferroptosis-induced alterations in intercellular communication. Ferroptosis has emerged as a promising target for controlling inflammation and impeding bone resorption in periodontitis.

## Materials and methods

### Murine experimental periodontitis model

C57BL/6 mice (7–8 weeks, male) were purchased from GemPharmatech (Guangdong, China) and bred in specific pathogen-free units with controlled room temperature and humidity and a 12 h light/dark cycle at the Sun Yat-sen University animal facility. Mice were cohoused in individual cages (maximum *n* = 5 per cage) during the experiment. All the experimental procedures were approved by the Institutional Animal Care and Use Committee of Sun Yat-Sen University (No. SYSU-IACUC-2023-001290). For the establishment of experimental periodontitis, the mice were first anaesthetized with isoflurane. A sterile 5–0 silk ligature was tied around the subgingival region of the maxillary first molar on the left side. The ligature was retained for 10 days. For drug administration, ligature-treated mice were allocated randomly into three groups: the Lip-1 group, the erastin group, and the vehicle group. Once the ligature was placed (Day 0), 10 mg/kg Lip-1 (T2367, TargetMOI), 30 mg/kg Erastin (T1765, TargetMOI), or an equal volume of vehicle was injected intraperitoneally once every other day over the 10-day period. Vehicle for Lip-1 and erastin: 5% DMSO, 40% PEG300, 5% Tween 80, and 50% ddH2O. All the mice were euthanized on Day 10. Maxillae and gingiva samples were then collected.

### Micro-CT analysis

Micro-CT scanning was performed with a Scanco Medical μCT 50 System (Scanco Medical AG, Bassersdorf, Switzerland) at a 10 μm resolution for quantitative analysis. The trabecular bone between the distobuccal root of the upper first molar and the mesiobuccal root of the upper second molar that extended from the coronal bone surface to 300 μm apically was selected as the ROI for bone parameter analysis, including bone volume over total volume (BV/TV), trabecular number (Tb.N), trabecular thickness (Tb.Th), and trabecular spacing (Tb.Sp). The CEJ-ABC distance was measured at six different sites on the buccal and palatal side (distal, middle, and mesial) via three-dimensional reconstruction using MIMICS 17.0 (Materialize, Leuven, Belgium). The mean CEJ-ABC distance of each mouse was used for statistical analysis.

### Histological staining and immunohistochemistry

The mouse maxillae were dissected, fixed in 4% paraformaldehyde for 48 h, and decalcified in 0.05 M EDTA for 4 weeks. After dehydration, the specimens were embedded in paraffin and sectioned at 5 μm along the sagittal plane of the upper first molar. For morphological analysis, sections were stained using an H&E staining kit (Servicebio, Wuhan, China). Osteoclasts were visualized by TRAP staining (387A, Sigma–Aldrich, USA) according to the manufacturer’s instructions. For immunohistochemistry, antigen retrieval was performed with 20 μg.mL^−1^ proteinase K (Sigma‒Aldrich) at 37 °C for 1 h after deparaffinization and rehydration. The sections were incubated with an anti-4-HNE adduct antibody (bs-6313R, 1:200, Bioss) overnight at 4 °C. HRP-linked secondary antibody (ab205718, 1:10000, Abcam) and a DAB detection kit (GeneTech, Shanghai, China) were used the next day according to the manufacturers’ instructions. Images were captured with a slide scanner (Leica Aperio AT2, Nussloch, Germany).

### TUNEL assay

Cell death was assessed using a DeadEnd™ Fluorometric TUNEL System (Promega, Wisconsin, USA) according to the manufacturer’s instructions. Briefly, for analysis of sagittal sections of the upper first molar, the slides were treated with proteinase K (20 μg.mL^−1^) at 37 °C for 1 h after deparaffinization and rehydration. For analysis of adherent cells, treated cells were cultured on glass bottom dishes, fixed with 4% formaldehyde, and permeabilized with 0.2% Triton X-100. Next, the tissues or cells were incubated with TdT reaction mix at 37 °C for 1 h and counterstained with DAPI solution. Dead cells with green fluorescence were detected by confocal fluorescence microscopy (Olympus FV3000, Tokyo, Japan).

### Quantitative real-time polymerase chain reaction

For the extraction of tissue RNA, the gingivae surrounding the maxillary first molars of the experimental mice were isolated and ground in TRIzol (Life Technologies, USA) using an automatic fast grinder (JXFSTPRP-24, Shanghai Jingxin, China). Total RNA was then extracted with a TRIzol-based method. Total RNA from MLO-Y4 cells was isolated using an RNA-Quick Purification Kit (Yishan Biotechnology, China). The extracted RNA was reverse transcribed into cDNA using PrimeScript^TM^ RT Master Mix (Takara, Japan) following the manufacturer’s protocol. RT‒qPCR was performed with Hieff qPCR SYBR^®^ Green Master Mix (Yeasen, China) on an ABI QuantStudio5 system. The relative mRNA expression of genes was determined by the 2^−ΔΔCT^ method and normalized to β-Actin expression. The primer sequences are listed in Supplementary Table S1.

### Immunofluorescence staining and confocal microscopy

For immunofluorescence staining, antigen retrieval was performed with proteinase K (20 μg.mL^−1^) at 37 °C for 1 h on paraffin sections. Primary antibodies against the following proteins were used: DMP1 (DF8825, 1:100, Affinity), GPX4 (ab231174, 1:100, Abcam), TfR1 (13-6800, 1:100, Invitrogen), 4-HNE (bs-6313R, 1:200, Bioss), RANKL (AF0313, 1:100, Affinity), and RUNX2 (12556 S, 1:100, Cell Signaling Technology). An M.O.M. kit (Vector Lab, USA) was used for the anti-TfR1 antibody. Alexa Fluor-conjugated secondary antibodies (Invitrogen) were applied for fluorescent labeling. Images were captured with a confocal fluorescence microscope (Olympus FV3000, Tokyo, Japan).

### Cell culture

The MLO-Y4 cell line was purchased from Zhong Qiao Xin Zhou Biotechnology Co., Ltd. (Shanghai, China), authenticated by STR profiling, and tested negative for mycoplasma contamination. The cells were cultured in α-MEM supplemented with 10% FBS and 1% penicillin/streptomycin at 37 °C in a humidified atmosphere of 5% CO_2_. For drug administration, cells were treated with vehicle (0.1% DMSO), *P.g.*-LPS (10 μg/ml, InvivoGen, USA), or Lip-1 (1 μM) + *P.g.*-LPS (10 μg/ml) for 24 h (RT‒qPCR) or 48 h (other analyses). In the Lip-1 + *P.g.*-LPS group, the cells were incubated with Lip-1 for 1 h prior to treatment with LPS.

### CCK-8 assay

Cell viability was analysed by a CCK-8 assay (CK04, Dojindo). Briefly, MLO-Y4 cells were treated as indicated for 48 h and then incubated with CCK-8 solution at 37 °C for 1 h. The absorbance (450 nm) of each well was measured by a microplate reader (BioTek Synergy H1, Vermont, USA).

### Measurement of intracellular ROS and lipid peroxidation

To analyse intracellular ROS, cells were treated as indicated and incubated with fresh serum-free medium containing 10 μM DCFH-DA (Beyotime, China) at 37 °C for 20 min. Fluorescence at 488/525 nm was detected using a multimode microplate reader (BioTek Synergy H1). To analyse lipid ROS, cells were cultured on glass bottom dishes in advance and incubated with 10 μM C11-BODIPY (D3861, Invitrogen) at 37 °C for 30 min. Fluorescence images were captured with a confocal fluorescence microscope (Olympus FV3000) at 581/591 nm for the reduced state and 488/510 nm for the oxidized state.

### Western blot

Treated cells were lysed in RIPA buffer supplemented with protease inhibitors for protein extraction via ultrasonication. The protein concentration was determined by a BCA assay kit (CWBIO, China). The following primary antibodies and concentrations were used for western blotting: GPX4 (ab231174, 1:1000, Abcam), TfR1 (13-6800, 1:1000, Invitrogen), RANKL (ab9957, 1:1000, Abcam), OPG (ab183910, 1:1000, Abcam), IL-6 (A24522, 1:800, ABclonal), GAPDH (2118, 1:1000, Cell Signaling Technology), and β-Actin (4967, 1:1000, Cell Signaling Technology). The secondary antibodies were HRP-conjugated, and visualization was performed with a ChemiDoc Imaging System (Bio-Rad, California, USA). Full-length uncropped western blots are uploaded in the supplementary material.

### Statistical analysis

All analyses were performed using GraphPad Prism (version 9.1, USA). No statistical methods were used to determine sample size. No samples or animals were excluded from the analysis. No blinding was done during the experiment. The data are presented as the means ± standard deviations. All data were tested for normality and homogeneity of variance. Unpaired Student’s *t* test was used to compare means between two groups. For significant differences among groups, one-way ANOVA was applied with Bonferroni correction for multiple comparisons. All tests were two-sided and differences were considered significant when *P* < 0.05. All experiments were performed in biological triplicates.

### Supplementary information

Supplementary material

Full-length uncropped western blots

## Data Availability

All data within the text and Supplementary Material are available from the corresponding author upon reasonable request.
