# Supplementary material for: Unraveling ferroptosis in osteogenic lineages: implications for dysregulated bone remodeling during periodontitis progression
Source: Cell Death Discov. 2024 Apr 26;10:195. doi: 10.1038/s41420-024-01969-6 (PMC11053120; doi:10.1038/s41420-024-01969-6)
Supplement: Supplementary file 1 — Supplementary material [file 41420_2024_1969_MOESM1_ESM.docx]

**Supplementary Material**

**Unravelling Ferroptosis in** **Osteogenic Lineages: Implications for Dysregulated Bone Remodelling during Periodontitis Progression**

Yiqi Tang^1^#, Sihui Su^1^#, Rongcheng Yu^1^, Chenxi Liao^1^, Zhili Dong^1^, Chengyao Jia^1^, Vicky Yau^2^, Liping Wu^1^*, Weimin Guo^3^*, Jinxuan Zheng^1^*

^1^ Hospital of Stomatology, Guangdong Provincial Key Laboratory of Stomatology, Guanghua School of Stomatology, Sun Yat-sen University, Guangzhou, 510055, PR China

^2^ Department of Oral and Maxillofacial Surgery, University at Buffalo, Buffalo, New York 14214, USA

^3^ Department of Orthopedic Surgery, Guangdong Provincial Key Laboratory of Orthopedics and Traumatology, First Affiliated Hospital, Sun Yat-sen University, Guangzhou, 510080, PR China

#These authors contributed equally.

*Corresponding authors

**Supplementary Figures S1-S2**

**Supplementary Table S1**

**
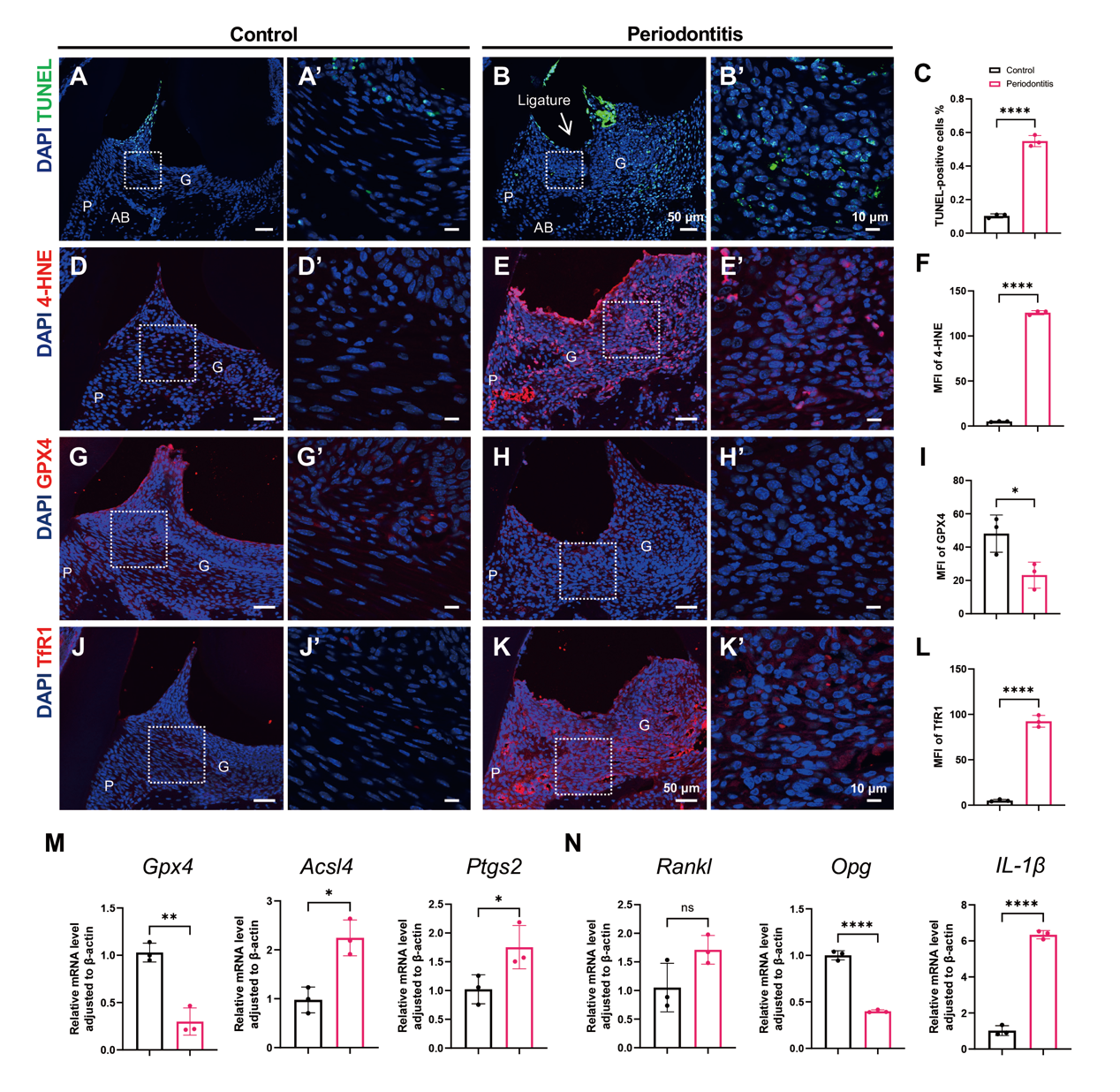
**

**Supplementary Fig. S1: Ferroptosis is induced in the gingiva of murine periodontitis models. A, B** Representative TUNEL staining images of dead cells in the distal gingiva of the upper first molar. The white arrow indicates the position of the ligature. Scale bar, 50 μm. **A’, B’** Magnified views of the boxed areas in Panels A, B. **C** The percentage of TUNEL-positive cells in the gingiva was quantified. Scale bar, 10 μm. **D, E** Representative immunofluorescence images of 4-HNE expression in the distal gingiva of the upper first molar. Scale bar, 50 μm. **D’, E’** Magnified views of the boxed areas in Panels D, E. Scale bar, 10 μm. **G, H** Representative immunofluorescence images of GPX4 expression in the distal gingival papilla of the upper first molar. Scale bar, 50 μm. **G’, H’** Magnified views of the boxed area in Panels G, H. Scale bar, 10 μm. **J, K** Representative immunofluorescence images of TfR1 expression in the distal gingival of the upper first molar. Scale bar, 50 μm. **J’, K’** Magnified views of the boxed areas in Panels J, K. Scale bar, 10 μm. **F, I, L** Quantification of the mean fluorescence intensity of 4-HNE, GPX4, and TfR1 in the gingiva. **M, N** Relative mRNA expression of *Gpx4*, *Acsl4*, *Ptgs2*, *Rankl*, *Opg* and *IL-1β* in the murine gingiva. G, gingiva; AB, alveolar bone; P, periodontal ligament. The data are shown as the means ± SDs; n = 3 mice; ns, not significant; **P* < 0.05; ***P* < 0.01; *****P* < 0.0001.

**
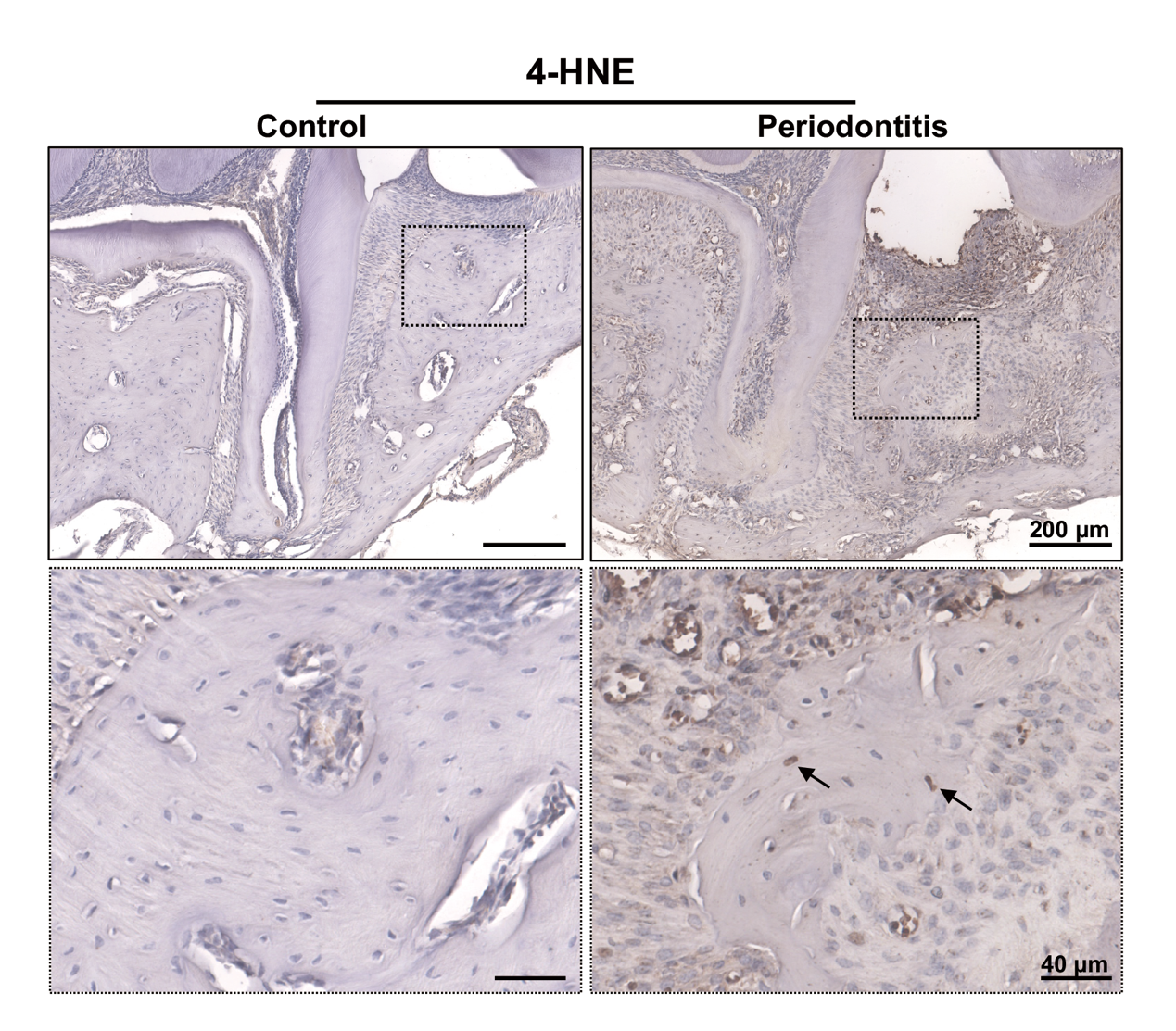
**

**Supplementary Fig. S2: Immunohistochemistry of 4-HNE in the alveolar bone of the periodontitis and control mice.** The lower panel shows magnified images of the boxed areas in the upper panel. Arrows indicate 4-HNE-positive osteocytes. Scale bar in the upper panel, 200 μm; Scale bar in the lower panel, 40 μm.

**Supplementary Table S1:** **Sequences of RT‒qPCR primers.**

| Genes | Forward (5'-3') | Reverse (5'-3') |
| --- | --- | --- |
| *Slc7a11* | AATACGGAGCCTTCCACGAG | CTCCAGGGGCAGTCAGTTAG |
| *Ncoa4* | GCCAGAGCAGAAGTCAGCAT | GTCCTGTGGGTTGGTACTGG |
| *Gpx4* | GTCTGGCAGGCACCATGT | GTGACGATGCACACGAAACC |
| *Acsl4* | GCACCTTCGACTCAGATCACA | GAAGCCAGCAATAAAGTACACAGA |
| *Ptgs2* | TCACGTGGAGTCCGCTTTAC | AGGATGCAGTGCTGAGTTCC |
| *Rankl* | TGAAGACACACTACCTGACTCCTG | CCCACAATGTGTTGCAGTTC |
| *Opg* | GGACCACAATGAACAAGTGGC | AACCCCTGCCTGAATCTTAGC |
| *IL-1β* | GCCACCTTTTGACAGTGATGAG | GACAGCCCAGGTCAAAGGTT |
| *β-actin* | TGTCCACCTTCCAGCAGATGT | AGCTCAGTAACAGTCCGCCTAGA |
