# Supplementary figures and images for: Unraveling ferroptosis in osteogenic lineages: implications for dysregulated bone remodeling during periodontitis progression
Source: Cell Death Discov. 2024 Apr 26;10:195. doi: 10.1038/s41420-024-01969-6 (PMC11053120; doi:10.1038/s41420-024-01969-6)

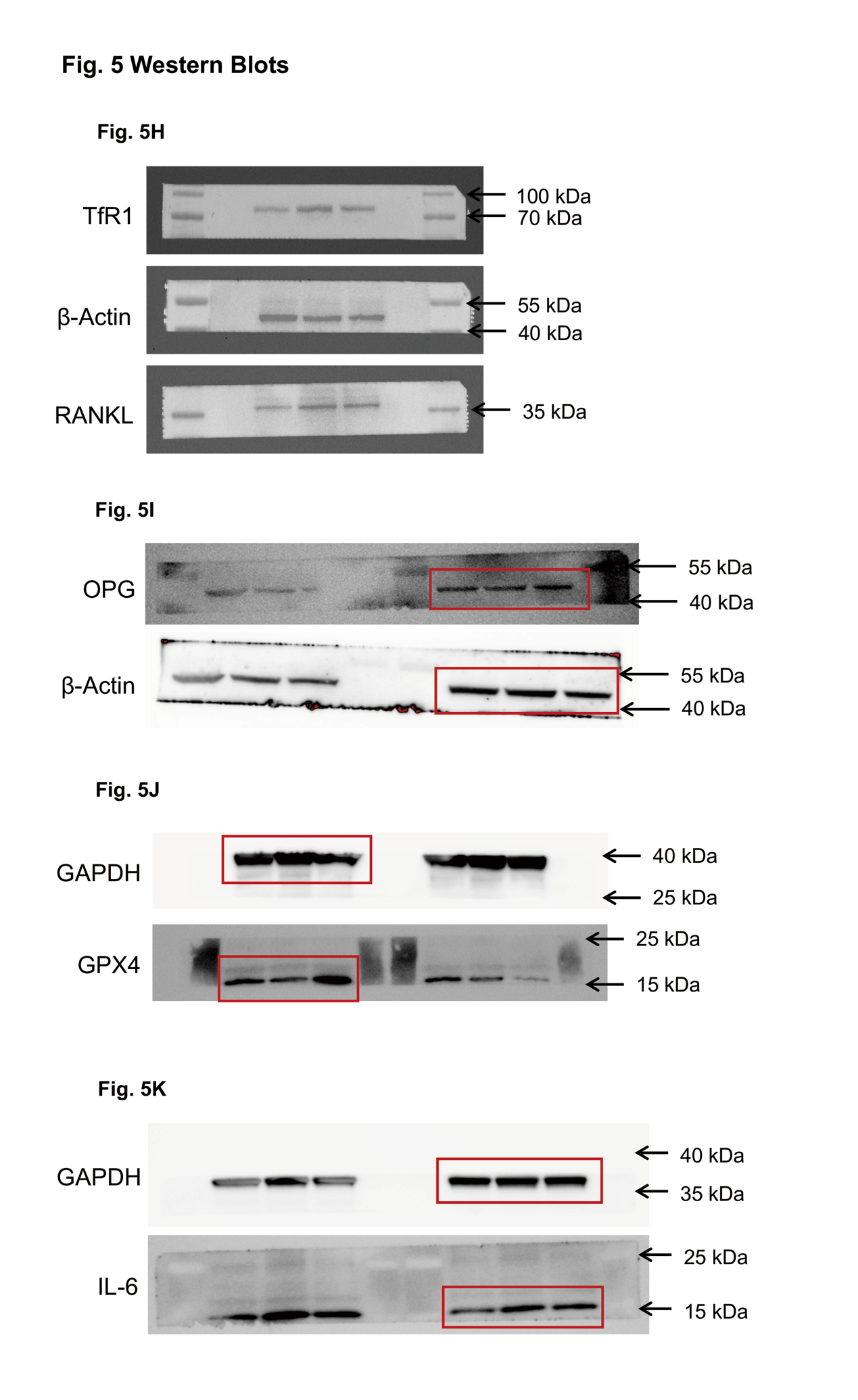


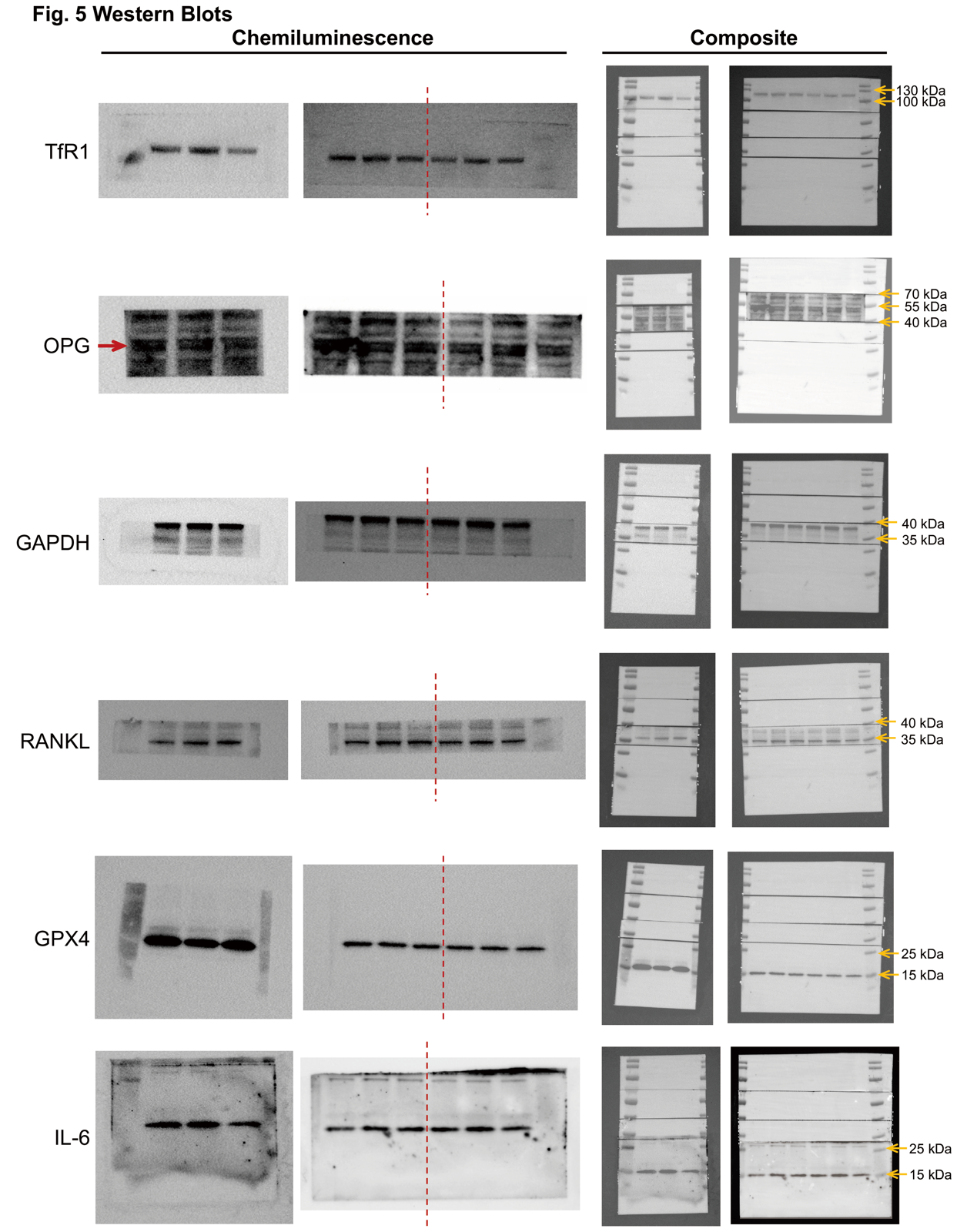


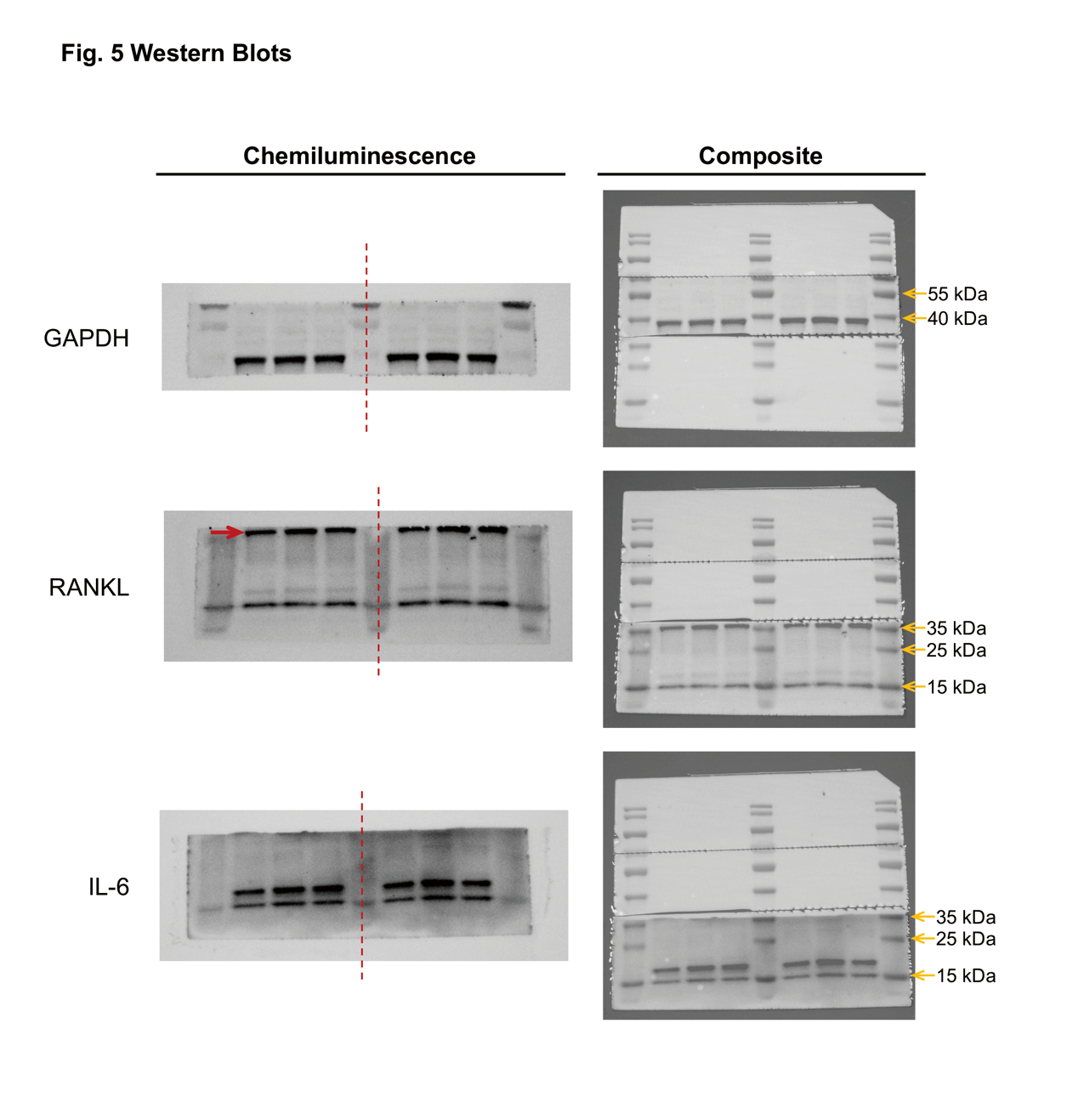


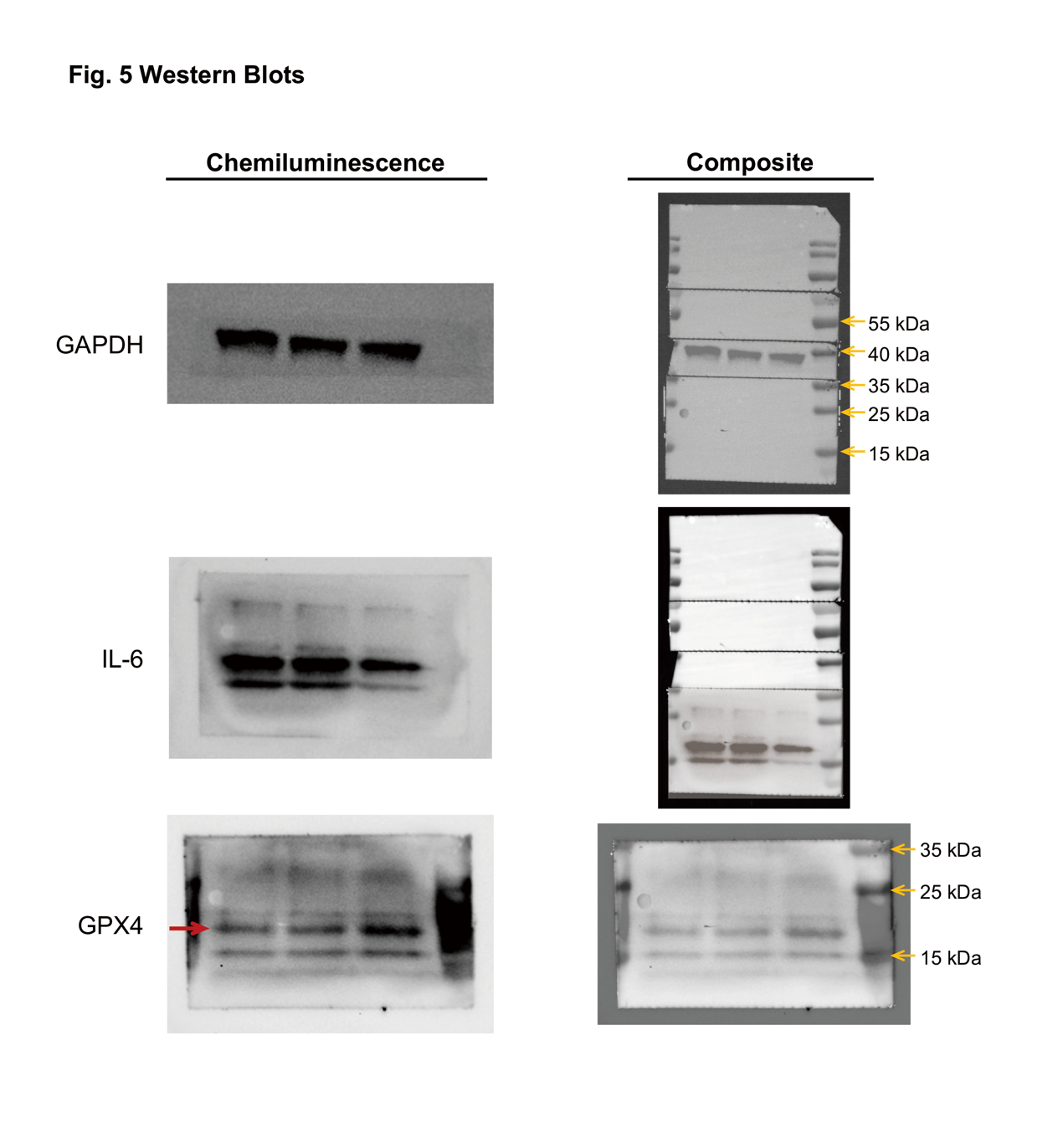

Supplement: Supplementary file 2 — Full-length uncropped western blots [file 41420_2024_1969_MOESM2_ESM.docx]
